# Autobiographical memory and hyperassociativity in the dreaming brain: implications for memory consolidation in sleep

**DOI:** 10.3389/fpsyg.2015.00874

**Published:** 2015-07-02

**Authors:** Caroline L. Horton, Josie E. Malinowski

**Affiliations:** ^1^Psychology, Bishop Grosseteste University, Lincoln, UK; ^2^Department of Psychology, University of Bedfordshire, Luton, UK

**Keywords:** autobiographical memory, dreaming, memory consolidation, sleep, continuity hypothesis, hyperassociativity

## Abstract

In this paper we argue that autobiographical memory (AM) activity across sleep and wake can provide insight into the nature of dreaming, and vice versa. Activated memories within the sleeping brain reflect one’s personal life history (autobiography). They can appear in largely fragmentary forms and differ from conventional manifestations of episodic memory. Autobiographical memories in dreams can be sampled from non-REM as well as REM periods, which contain fewer episodic references and become more bizarre across the night. Salient fragmented memory features are activated in sleep and re-bound with fragments not necessarily emerging from the same memory, thus de-contextualizing those memories and manifesting as experiences that differ from waking conceptions. The constructive nature of autobiographical recall further encourages synthesis of these hyper-associated images into an episode via recalling and reporting dreams. We use a model of AM to account for the activation of memories in dreams as a reflection of sleep-dependent memory consolidation processes. We focus in particular on the hyperassociative nature of AM during sleep.

## The Context of Autobiographical Memory

Whilst memories for very specific instances are referred to as “episodic” (e.g., Tulving, 1983, 2002), memory and information for and about our own experiences more generally falls under the umbrella term of “autobiographical memory” (AM; Conway and Pleydell-Pearce, 2000; Conway, 2001, 2005, 2009; Conway and Loveday, 2015). AM is a representation of experiences (episodic memories and associated details) and information (semantic knowledge) related to the self. As such it engages all aspects of the declarative memory system (Nadel, 2008; Renoult et al., 2012). Current conceptualizations of episodic memory, which break from earlier theories (e.g., Tulving, 1983), suggest that episodic memories are detailed summary accounts of short time-period experiences, which are forgotten within 24 h unless they are consolidated during sleep and subsequently become linked to AMs (Conway, 2009). Life stories, or autobiographies, are created over time into a narrative, compiled of personally salient, emotional and everyday experiences, along with facts and knowledge about ourselves (Conway and Pleydell-Pearce, 2000). Similarly, dreams are narrative simulations of autobiographical episodes (Montangero, 2012), and it has been suggested that narratives are the “basic manner in which the brain organizes experiences” (Pace-Schott, 2013, p. 2)—hence both waking AM and sleep mentation are organized in this manner. Crucially, such narratives are not composed of precise replicas of specific (episodic) experiences from the life-course. Rather, they are constructed and changed over time, much as an author may edit a novel in order to improve its readability and clarity. We propose that AM functions comparably across sleep and wake (Horton, 2011a,b; Malinowski and Horton, 2014a).

We use this account of AM to describe and explain the construction of dreams. Furthermore we explore the processes of sleep-dependent memory consolidation with a particular emphasis on the enhancement, stabilization, and integration of AMs. In this paper we propose a model in which AM experiences are broken down into constituent fragments, reactivated “offline” during sleep, and re-combined via hyperassociativity (e.g., Llewellyn, 2013) into a novel experience. This gives rise to dreaming. We argue that these processes of sleep-dependent memory consolidation crucially rely on AM functioning, in contrast to alternative models, which do not address the multi-modality of memories. We emphasize we constructive nature of AM retrieval, recognizing (i) that memories change considerably over time, and (ii) that sleep can enhance AM by breaking down and re-combining memory elements, re-activating salient features and thus consolidating them via repeated activation. As such sleep-dependent AM consolidation functions to de-contextualize information, rendering salient features more retrievable following a period of sleep. In this paper we outline each of these processes in critical detail.

We will begin by reviewing the activation of AM across sleep as well as waking periods, exploring the inclusion of AMs in dreams. We then explore the activation of AM fragments in sleep and how they are hyperassociated. Next we examine the relevance of such hyperassociativity to processes of sleep-dependent memory consolidation, and finally propose a model of AM consolidation, which dreams can usefully reflect.

## Constructive Memory

The constructive and malleable nature of AM renders it notoriously error-prone. Memories for personal experiences may change over time in relation to personal goals (Conway, 2001), so to align with current conceptions of the self (Conway and Pleydell-Pearce, 2000) or as some function of decreased emotional reactivity (Walker and Skowronski, 2009; Ritchie et al., 2014). In terms of the latter, the Fading Affect Bias describes the reduction of negative emotion associated with an AM over time and has been evidenced in dreams as well as waking events (Ritchie and Skowronski, 2008). Furthermore rich, false episodic memories about one’s past are easily induced (Shaw and Porter, 2015). Conway and Pleydell-Pearce (2000) argue that this fluid autobiography operates under a self-memory system, in which an executive function of current personal goals filter the retrieval of long-term knowledge and experiences about one’s own life. As such, retrieving autobiographical experiences is an active process and reflects the flexibility of the cognitive systems underpinning it.

Such a dynamic view of recall is not often acknowledged, particularly in the memory consolidation literature. This is perhaps not surprising, given that exploring the changes in AM over time is resource-burdensome and methodologically challenging. Nevertheless, this fluid account of AM acknowledges involvement of both semantic (personal knowledge) and episodic systems within declarative memory (Renoult et al., 2012), and therefore may align well with more recent models of memory consolidation (see Memory Consolidation in Sleep) and reconsolidation (Alberini and Taubenfeld, 2008).

As opposed to AMs, distinctions between episodic and semantic memory systems have instead dominated experimental paradigms as they are much more easily manipulated, despite the presentation of word lists as representative of episodic experiences being low in ecological validity. Nevertheless, an understanding of the processes underlying AM construction—and consequently an understanding of the ways in which humans make sense of their own life stories and selves—relies in part upon an understanding of both episodic and semantic memory processes. (For a comparison between AMs and EMs see Conway, 2009.) The key processes are encoding, storage and retrieval. Much attention has been focused in recent years on storage as an active process of consolidation and re-consolidation, and it is this that we consider in this paper, in particular in terms of how sleep can enhance AM consolidation.

## Incorporations of Memories into Dreams and the Continuity Hypothesis

One’s autobiography is made up of experiences from across the lifespan also including thoughts, concerns and dreams. We argue that AM operates across all states of consciousness, with memories being accessible at times involuntarily (e.g., Berntsen, 1996). The characteristics of autobiographical recall (Conway, 2005) typify the flexible and fluid nature of memory retrieval, which is often overlooked in traditional experimental paradigms.

An extension of this view is that AM operates “offline,” during sleep and all its composite stages. Indeed this reflects the “continuity hypothesis” (Hall and Nordby, 1972; Schredl and Hofmann, 2003; Domhoff, 2011; Schredl, 2012; see also Horton and Malinowski, 2011); most broadly that there is overlap between cognitive processing (amongst other things) across sleep and wake. Most notably, such overlaps can be measured by comparing various aspects of (memories for) dreams to (memories for) waking experiences. A traditional view is that dreaming, and thus dream memory, is somehow deficient in comparison to waking memory (Crick and Mitchison, 1983). However as recalling both dreams and waking memories engage the same autobiographical system, some studies have demonstrated considerable overlap in the retrievability of dreams and waking experiences (Botman and Crovitz, 1990; Kemp et al., 2003; Grenier et al., 2005; Horton and Conway, 2009; Parke and Horton, 2009; Horton, 2011a,b, 2014, see also Graveline and Wamsley, 2015). Furthermore differences between waking AMs and dreams may result from methodological challenges, rather than actual differences. That is, waking AM is not flawless or even particularly accurate, although it is often deemed to be superior in accuracy and recallability than dream memories (see Chapman and Underwood, 2000). Unfortunately direct comparisons cannot be made between dream memories and waking AMs, as the validity of systematically sampled AMs can be verified whereas memories for dreams are the closest to an actual dream experience one can sample.

Nevertheless there is a wealth of evidence showing the continuity of AM across sleep and wake in terms of content (e.g., Stickgold et al., 2000; Schredl and Hofmann, 2003; Schredl et al., 2006), and consciousness and cognition (e.g., Graveline and Wamsley, 2015; Kahan and LaBerge, 1996, 2011; Kahan et al., 1997). As such the sleeping brain can reflect one’s personal life history to a similar extent as waking life processes and behaviors can. One notable method for exploring the presence of AMs during sleep involves measuring the incorporation of waking AMs into dream reports.

Three overarching mechanisms have been consistently identified to operate to determine the likelihood of a waking experience appearing in dreams: its emotionality (intensity as opposed to valence), its age (see Section “Is Dreaming a Reflection of the Processes of Sleep-Dependent Autobiographical Memory Consolidation?” on the day residue and dream-lag effects), and its personal salience. Additionally there seem to be further mechanisms that underpin the likelihood of those experiences appearing as a more holistic representation or a fragmentary one, and across different periods of sleep:

(i) Emotionality: Generally dreams are more emotional than waking memories, and this effect remains when recall bias is controlled for (Schredl and Doll, 1998; Malinowski and Horton, 2014b). This may result from the preferential incorporation of emotional AMs into dreams (Horton et al., 2011) as well as the heightened activity of the limbic system during (rapid eye movement, REM) sleep. In some cases intense and negative memories appear in dreams episodically, under conditions of post-traumatic stress (e.g., Hartmann, 1996).
(ii) In only very rare cases do truly episodic memories feature in dreams (Fosse et al., 2003; Schwartz, 2003; Malinowski and Horton, 2014a).
(iii) Self-representation: current self-images have been shown to be incorporated into dreams, reflecting the activity of the working-self (Horton et al., 2009); part of the self-memory-system model of AM functioning (Conway and Pleydell-Pearce, 2000; Conway, 2005).
(iv) More generally the number of self, semantic and episodic references vary across sleep-stage (see Foulkes et al., 1989), with each aspect appearing more fragmentarily, manifesting as increasingly bizarre, across the night (Hobson et al., 2000; Malinowski and Horton, 2014c). Concurrently the extent to which waking-life references appear in the dream in an abstract (opposing literal) way increases both across the night (Wamsley et al., 2010a), and over longer time periods (Blagrove et al., 2011b).
(v) Personally salient experiences may be preferentially incorporated (Malinowski and Horton, 2014b) and the age of these memory traces may be somewhat predictable (van Rijn et al., 2015).

The non-episodic manifestation of waking AMs into dreams has been well noted (as based on Fosse et al., 2003; Malinowski and Horton, 2014a). Fosse et al. (2003) originally noted that episodic “replay” of waking life experiences was so rare in dreams that there must exist a functional dissociation between REM dreaming and episodic memory. Rather than truly episodic experiences being “replayed,” as we would experience the recollection of an episodic memory during waking, elements of AMs feature heavily in dreams (Malinowski and Horton, 2014a), and may reflect the relative attenuation of frontal systems during (all stages of) sleep. According to Malinowski and Horton (2014a) whole, intact episodic memories are not useful information, therefore they are not replayed in dreams in this manner. We propose here an alternative function of AMs appearing in a more fragmented fashion during sleep: for salient fragments to be activated in a different combination, thus consolidating those features to improve their future retrievability in any context or situation that necessitates them.

Although autobiographical elements from waking life appear in dreams, experiencing a dream is often comparable to experiencing or thinking about something in the present. That is, the ability to reality monitor, or identify that a dream is a dream as distinct from waking life perception, is attenuated (unless experiencing lucidity). The dreamer typically engages with the dream-environment from a first-person perspective, is capable of thinking (McNamara, 2000), feeling (e.g., Schredl and Doll, 1998) and a host of other sensory experiences (Horton and Conway, 2009). Consequently measuring the apparent activation of fragmentary AMs in dreams seems incongruous with the fluid experience of dreaming, which is continuous, holistic and not unlike waking consciousness. Reconciling these views involves appreciating (i) that AM fragments may not be the sole constituents of a dream, (ii) that AM fragments are bound together into holistic narrative experiences (Montangero, 2012; Pace-Schott, 2013) giving rise to a multi-sensory experience, and (iii) that some cognitive processes engaged during waking may result in the synthesis of fragmentary dream elements at the point of recalling and re-telling a dream (see Horton, 2014). Indeed many models of consciousness recognize this quality of unifying disparate cognitive mechanisms and features (e.g., Baars, 2002).

Consider the following example of a dream recorded by a participant in our study (Malinowski and Horton, 2014a). The participant dreamt that she was in a back garden (of the house she grew up in), with a man (who was a character from the TV series “Heroes” which she had been watching), and he was choosing a bottle of wine (which was an activity she herself was used to doing). She later went into the kitchen and talked to a beautiful woman (who she believed to be a happier, more stable version of herself). This dream incorporates various elements of autobiographical information, both from the past (the childhood home) and arguably the future (the woman that she would like to become). Fragments of information about characters, the setting, the conversation, *et cetera*, have been taken from waking life from across a range of time-points, and have been re-bound into a novel experience. This differs substantially from experiencing a dream in which whole waking life episodic memories feature, as that would involve the characters, settings and conversations (for instance) to play out together in exactly the same way as they had been experienced during waking (Horton and Malinowski, 2011).

There is evidence that memory de-fragmentation (Payne et al., 2008b) and re-binding (Payne et al., 2009) occurs during sleep and that autobiographical recall processes further encourage the synthesis of fragmentary dream elements upon morning awakening (Hobson and McCarley, 1977; Horton, 2014). This further emphasizes the continuity of the AM system across functioning across sleep and wake.

We next propose that the appearance of re-bound AM elements in dreams is functional: to de-contextualize the salient features, as part of a process by which information can be identified as salient (in line with Stickgold and Walker, 2013). We return to this explanation in Section “Memory Consolidation in Sleep.”

## Hyperassociativity and Bizarreness

We argue that the process by which AM elements are re-bound in dreams occurs as a result of the hyperassociative nature of sleeping cognition (Stickgold et al., 1999; Llewellyn, 2013). Hyperassociativity, whilst rarely explicitly defined, refers to the increased activation of weakly semantically related concepts and networks, following the activation of a specific concept or memory (Stickgold et al., 1999). It therefore concerns a form of cognitive processing and has been widely proposed to illustrate typical processing within dreaming and, specifically, REM sleep (Antrobus, 1993; Hartmann, 1996; Stickgold et al., 1999; Hobson, 2002; Cai et al., 2009; Levin and Nielsen, 2009; Walker, 2009; Walker and Stickgold, 2010; Llewellyn, 2013). Specifically, the connectivity between associations can be made between memories that would be considered loosely associated during waking. Hartmann (1996, 2010a,b) argues that the associativity between activated memories can be modeled on a continuum with focused, waking thought at one end, increased associativity during daydreaming or meditation, for instance, and loose, or hyper, associativity featuring during dreaming. Hartmann (1996) proposes that the function of dreaming hyperassociativity is to process and weave emotions into more stable networks of prior experience, and we review this view elsewhere (see Malinowski and Horton, submitted). Whilst we recognize that such emotional memory assimilation during sleep is an important function of sleeping cognition (Malinowski and Horton, submitted), we recognize that hyperassociativity contributes to the selective processing of other, emotionally-neutral memories, also.

The function of this could be to allow specific fragments of waking experiences to be selectively reproduced, perhaps played out in a novel or bizarre context, rendering them context-free and subsequently increasing their inter-relations with other, more loosely-associated memory fragments. EMs, which are by definition context bound, engage hippocampal regions. AMs, instead, can be context-free and the advantage of this over time is that previously learned information is easily retrieved in any context, facilitating accessibility. Hyperassociativity involves the activation of information via a semantic network (e.g., McClelland and Rumelhart, 1985). The activated memories may be elements of experiences and may trigger the recollection of a full EM, though this is unlikely given (i) the loose and therefore disorganized nature of hyperassociativity, (ii) the lack of frontal activity during sleep, and (iii) the typical transfer of memories from hippocampal to cortical regions during sleep (Squire and Alvarez, 1995; also see Memory Consolidation in Sleep).

As noted previously, the selective fragmentation of memory elements during sleep followed by the re-binding of memory elements give rise to a holistic experience of dreaming, despite the re-bound elements often being disparate, implausible or impossible. In one study, Montangero (2012) found that 81% of dreams contained abrupt and complete changes of dream events, concluding that dream content has a “coherent but unexpected” (p.162) nature, alluding to the disparate yet narrative structure of dreams. Such odd or novel associations result from hyperassociative processing and some authors argue that this gives rise to positive experiences of insight, creativity and problem solving either during or immediately following a dream (see Barrett, 2012, for a summary). As such the hyperassociative nature of sleeping cognition could account for the discontinuities of form, context, time or location that are often reported in dreams, more commonly referred to as “bizarreness.” Indeed it has been suggested that bizarreness arises out of the joining together of disparate elements (often from waking life; e.g., Revonsuo and Tarkko, 2002; Levin and Nielsen, 2007), which is also indicative of hyperassociativity.

## Potential Functions of Activated Memory Fragments

Whilst some theorists argue that the creative insight gained from the novel arrangements of familiar stimuli within dreams might be a function of dreaming, there is an additional growing consensus and body of evidence implicating the role of memory, in particular improvements in recall, in relation to sleep and dreaming. These two potential functions of dreaming may be related. Given the inextricable links between AM and dreaming, we consider the AM system to be instrumental in the selective consolidation of memory during sleep, and in the associated formation of dreams. The fragmentation of AMs during sleep and subsequent re-combination of these (in dreams) not only provides novel arrangements and potential insight, but also involves the re-activation of salient features, thus consolidating those elements. We draw on other models of fragmented memory during sleep/dreaming to inform the development of our own model.

Levin and Nielsen (2007, 2009) and Levin et al. (2010) proposed the AMPHAC/AND model of dreaming. The acronyms refer to the neural circuits involved in the activation of relevant memories, emotional in this case, and their consequential fear-extinguishing function. The model accounts for the fragmentation of AM elements and their recombination into novel permutations, as described above, though with the emphasis on playing out emotional elements in a new context so to reduce the associated negative emotion (almost always fear). This theory accounts for the bizarreness so typical of dreams, the regular emotional content of dreams (Schredl and Doll, 1998) and the reduction of emotional intensity of repeated dream sources over time (e.g., see Cartwright, 2010, for a summary; Walker and van der Helm, 2009). The theory is therefore comprehensive and strongly supported by empirical data linking emotion regulation, processing, and dreaming (see Malinowski and Horton, submitted, for a thorough review). However, there are some aspects of sleep-dependent memory processing that the model does not account for, namely the links between sleep and memory consolidation, and the activation of positive as well as negative (non-fear-based) emotions (Schredl and Doll, 1998). Thus the AMPHAC/AND model acknowledges some of the trends of memory fragmentation and re-binding that occur during sleep, but offers an explanation for this based on emotional-processing rather than broader memory activation and consolidation.

There is a great wealth of evidence implicating emotional arousal as a mediator of the sleep-memory relationship (see Malinowski and Horton, submitted, for a review). As such Levin et al. (2010) emphasis upon this variable is appropriate, Future models need to acknowledge the role of emotionality in sleep-dependent memory consolidation in healthy, as well as clinical, populations. The previously mentioned Fading Affect Bias (e.g., Ritchie et al., 2014), in which the negative affect associated with a memory fades over time, was demonstrated for dream elements (Ritchie and Skowronski, 2008), reflecting further continuity in AM over sleep and wake as well as the fragmentation of AMs in dreams. The de-coupling of AMs from their emotional context has been demonstrated elsewhere (e.g., Payne et al., 2008b) and may indicate one of at least two possible mechanisms: firstly that emotional arousal associated with an AM is a marker of its salience, thus its need for consolidation; secondly that the associated emotional arousal clouds the consolidation process, thus extracting the negative affect from the remaining AM purifies the remaining elements for subsequent processing. A possible secondary process concerns the addition of associated positive affect with an AM over time, as part of the constructive recall process, in which experiences are retrieved in line with current conceptions of the self (Conway and Pleydell-Pearce, 2000).

Whilst the fading affect bias exemplifies the continuity of AM across sleep and wake, it does not propose a specific model by which these relationships exist. We next consider a memory-based account of memory activation in sleep and dreams and bear in mind the likely mediating role of emotion.

Johnson (2005) proposed that the content of dreams reflects memory activation which serves the purpose of developing “context memory.” Johnson argued that information is initially learned within a specific context and then later decontextualized. This is hypothesized to occur over the life-course, with the apparent need for more REM sleep at birth taken as being supportive of this claim. There are three main limitations to this view. Firstly context memory is defined extremely vaguely and never operationalized. Context memory may refer to relational memory or sematic-association networks that pervade other literature and cognitive models (e.g., McClelland and Rumelhart, 1985), but this is unclear. Secondly Johnson draws parallels between REM sleep and dreaming, which is a largely-redundant and out-dated idea, given the wealth of evidence that dreaming can be sampled from non-REM periods (e.g., Foulkes et al., 1989) and that REM does not always give rise to dreaming (e.g., Solms, 2000). Thirdly, whilst we might indeed initially learn of experiences in a context-dependent manner, it may not be advantageous to do so. Take for instance the experience where we cross a road and have an unnervingly close encounter with a speeding car. It would be logical to extract and consolidate the elements of that experience on the basis of future need. In that case, that vehicles (not just cars) may pose a threat to our safety. As such there is a need to retrieve this useful information and apply it to future road-crossing scenarios, i.e., by being careful and attentive of oncoming vehicles. According to Johnson’s theory, the context of road-crossing could pose a general threat and that context-bound detail would be consolidated during REM sleep. However, evidence shows that emotional aspects of visual memories (scenes) can be decoupled from their broader contexts and selectively consolidated during sleep (Payne et al., 2008b). So experiences can be broken down into fragments, each perhaps evaluated on the basis of emotionality or arousal, and activated individually. This move away from episodic consolidation aligns not only with our proposals concerning the fragmentation of autobiographical, rather than episodic, experiences (see, also Malinowski and Horton, 2014a) but also broader empirical illustrations of learning showing the “R-K shift”—a move from episodic to semantic retrieval of the same learned items over time (Dewhurst et al., 2009). Furthermore sleep-dependent memory consolidation processes generally propose that memories become less episodic and more semanticized, as reflected by their decreased hippocampus-dependency over time (Nadel and Moscovitch, 1997; Marshall and Born, 2007), and that the activation of more episodic memories tends to occur during slow wave (non-REM) stages of sleep. Thus Johnson’s ideas concerning context memory seem largely at odds with the evidence for sleep-dependent memory consolidation and the associated fragmentation of activated memories appearing in dreams.

To continue with the road-crossing example, we can assume that memory consolidation serves the purpose of enhancing the retrievability of important aspects of an experience. This would necessitate identifying and then extracting salient features of that experience. Subsequently this would break down the episode into smaller units, and as such de-contextualizing it. The salient features would be identified as such, either by being re-activated or being associated with arousal of some kind, forming part of the consolidation process. The result would be that consolidated memory fragments would be better integrated into pre-existing networks and being more easily retrieved in times of need. To continue with this example, the recollection that vehicles may pose a threat could be applied to other potentially threatening contexts. We therefore propose the opposite to Johnson (2005) in that functional de-contextualization and fragmentation of memory experiences leads to enhanced memory. In order to appreciate this, let’s briefly review our understanding of memory consolidation during sleep, then how that may relate to dreaming.

## Memory Consolidation in Sleep

Memory consolidation refers to the stabilization and integration of information into long-term memory networks (Marr, 1970). This may be measured either by an increase in performance in a memory task (enhancement) or a lack of a reduction in performance (maintenance). We focus here on consolidation at a systems-level of brain and cognition, rather than synaptic-level changes over time, as the systems level reflects the activation and engagement of different brain regions, which can provide insight into the cognitive processes involved in consolidation. The standard model of consolidation (Squire and Alvarez, 1995) proposes that memories become hippocampal-independent over time, with the time-course of this process varying depending on the complexity of the memory though ranging from hours to weeks. In contrast the multiple trace theory (Nadel and Moscovitch, 1997) emphasizes the distinct processing of different kinds of declarative memories, in that episodic memories always remain somewhat hippocampal-dependent whilst semantic memories become independent. We will briefly critique these in light of the processing of AMs here.

Our understanding of the time-course of [hippocampal– cortical] memory consolidation comes in part from cognitive neuropsychology, namely patient studies of amnesics, as well as from cognitive neuroscience and animal lesion studies. The latter show a sharper decline in temporally graded retrograde amnesia compared to humans, spanning up to weeks as opposed to years (see Frankland and Bontempi, 2005, for a brief review). According to the standard view there is coordinated activation of recent memories across hippocampal–cortical networks, which leads to a gradual strengthening of cortico-cortical connections. In turn these render all new memories independent from the hippocampus and to be gradually integrated with pre-existing cortical memories (Squire and Alvarez, 1995). A feature of this standard view is that such activation occurs by replaying the original experience in some form (Marr, 1970). The alternative, multiple trace theory, approach to systems consolidation emphasizes the different networks involved in processing episodic (and therefore detailed autobiographical) memories from semantic memory (Nadel and Moscovitch, 1997). This view draws on evidence that contextually-rich episodic and spatial information within memory almost always activates the hippocampus. Thus consolidation of these memories differs from the cortical networks involved in the retrieval of more semantic, or non-declarative, knowledge. The multiple trace theory therefore has weight in that it addresses the differences between context or experience-based memories, and knowledge, though adds a layer of complexity to understanding the processing of most AMs, which comprise both episodic and semantic elements (Renoult et al., 2012).

Whilst both accounts of systems consolidation imply that memories can be set down into relatively stable structures, it is widely acknowledged that such memories can be subject to change and refinement over time (Alberini and Taubenfeld, 2008). Indeed our comprehension of the AM system (e.g., Conway and Pleydell-Pearce, 2000) serves as a reminder that retrieved memories are rarely recalled truly episodically. Rather, fragments of information about an experience, perceptions of it and information gained since an experience will be mixed in with the original experience, leading to different kinds of memory integration and transformation, such as gist extraction and insight generation (Payne, 2010; Wamsley, 2014). In this way memories are ever-changing. This is likely true of all kinds of memories, such as procedural knowledge (learning how to drive a car for instance) in addition to autobiographical experiences, for which construction is already a well-documented feature. “Re-consolidation” refers to the processes whereby recalling a memory re-activates and slightly changes it, requiring it to be re-integrated into ever refined semantic structures (Nader, 2003). Thus the time-course of consolidation processes reflects the malleable nature of remembering. Consolidation is not instantaneous, which falls in line with this view. Rather it occurs over hours, days, weeks and perhaps even longer (see Frankland and Bontempi, 2005).

Sleep is largely seen to be the state during which most memory consolidation takes place (see Payne et al., 2008a, for a thorough review). During sleep external stimuli are not perceptible, unless salient or loud enough to rouse the sleeper. Here the brain enters a reflexive state in which thoughts and experiences are internally generated (e.g., Maquet, 2000). Thus consolidation allows for previously encoded and accessible memory traces to be activated in some form without the interference of new stimuli. Alternative theories of sleep-dependent memory consolidation such as Crick and Mitchison (1983) promote the idea of the brain requiring downtime in order to process and sort what has been perceived before, and that sleep provides the ideal environment for this to occur. However, considering sleep-dependent consolidation as an “offline” process hints at passivity, which is misleading. Rather, a current body of research highlights the selective activation and processing of salient features of memories and experiences. Identifying the kinds of memories selected for activation, and how they may be broken down into constituent parts can provide insight into the larger-scale processes of memory consolidation in the sleeping brain. In time this may lead to specific and perhaps predictable hypotheses concerning dream content, for example that dream content will reflect emotionally intense waking-life experiences (Malinowski and Horton, 2014b).

Researchers are beginning to question which specific neural circuits are strengthened during sleep, and which features of a memory are preferentially retained over time. Many researchers argue that emotional features of a memory are retained (e.g., Holland and Lewis, 2008). Others have found a more general personal salience effect (e.g., van Rijn et al., 2015). Recently sleep has been shown to enhance memory features expected to be of future relevance, with specific slow-wave sleep features (spindles and slow oscillation activity) being associated with preferential consolidation (Wilhelm et al., 2011). Similarly intended actions, or goals, are more likely to be carried out following sleep (Diekelmann et al., 2013). Taken together there appears to be at least two levels of selectivity involved: a basic level in which arousal at activation might indicate whether a memory feature is deemed sufficiently salient to warrant future processing, and a higher-level process in which some more personal method of selectivity is engaged, on the basis of individual need or assimilation into an autobiographical goal hierarchy. Further research is certainly required here, though Stickgold and Walker (2013) review and emphasize the active selectivity of consolidation processes in their “triage” theory, which acknowledges in part the higher-level processes. Lewis and Durrant (2011) propose a model of sleep-dependent consolidation of schematic information, or gist extraction. Here the activation of different memories during sleep leads to overlapping and therefore repeated activation of specific memory fragments, rendering those fragments subject to greater processing and consolidation. Whilst this view may seem contradictory to Stickgold and Walker’s (2013) view in that it is much more passive, with activation frequency determining consolidation, it may account for sleep-dependent processing at a later stage than the processes of selectivity outlined in the triage theory. This could, perhaps, occur later in the course of the night’s sleep.

Alternatively, both systems could operate in parallel and refer to episodic (Stickgold and Walker, 2013) and semantic (Lewis and Durrant, 2011) consolidation, respectively. We believe that memory consolidation involves both the strengthening of traces representing the episodic details of experience, and the parallel integration of information extracted from experience with previously acquired semantic knowledge. In this view, episodic experiences and semantic knowledge are simultaneously broken down for processing by the same overarching AM system. Hippocampal activity reflects the activation of unique representations of episodic experiences, while cortical activation reflects semanticized and integrated information. As AM involves the activity of both declarative memory systems, it requires the processing and consolidation of experiences episodically first and more semantically later, though some experiences always retain their episodic nature. There is evidence to suggest that emotional memories, in particular, retain such episodic features. We will re-visit this idea in Section “Is Dreaming a Reflection of the Processes of Sleep-Dependent Autobiographical Memory Consolidation?” when we consider the time-course of memory incorporation and activation in dreams.

## Contributory Roles of SWS and REM

Thus far we have focused largely on sleep-dependent memory consolidation as if sleep is a homogeneous state, although we have briefly referred to slow-wave-sleep in the preferential consolidation of episodic memories and REM in more semantic memory (Foulkes et al., 1989; Rauchs et al., 2005). REM has also been heavily implicated in the processing of emotional memories (Wagner et al., 2001). The profile of sleep across a typical night shows a greater density of slow-wave activity in the first half of the night, and more REM later. This may reflect the sequence of sleep-dependent memory processing. Generally, slow wave sleep involves the reactivation of experiences from the day, then those experiences are broken down and emotional, and/or salient aspects selectively processed, during REM.

Furthermore during non-REM, there is evidence for gist-extraction and schematic representation (Lewis and Durrant, 2011; Tamminen et al., 2013), with further evidence for problem-solving and insight benefitting from sleep compared to wake (Wagner et al., 2004), perhaps benefitting specifically from REM (Cai et al., 2009). These effects can be summarized as a de-contextualization of originally episodic experiences. During REM however the memory aspects that require future use and/or retrievability are selected according to some mechanism (Stickgold and Walker, 2013), re-bound with other fragments leading to novel permutations and creative insight, and are further consolidated. It is during these stages, and/or later in the night of sleep, that particularly emotional, important and future-relevant aspects of information appear preferentially in dreams (Malinowski and Horton, 2014b). We might assume, therefore, that emotional salience acts as a marker for activating memory fragments during REM sleep, that were decontextualized during the preceding non-REM stages of sleep, which together result in heightened retrievability of relevant memory fragments, ready for future autobiographical use (Malinowski and Horton, submitted).

Schwartz (2003) offers an explanation for the fragmentary appearance of EMs (as AMs) in dreams: as recent EMs are hippocampus-dependent (Marshall and Born, 2007), the information flowing from hippocampus to cortex during slow-wave sleep reflects the activation of more episodic information (engaging the hippocampus). However, during REM the information flow is blocked, with cortical-hippocampal flow increasing instead. Thus older, more semanticized memories (being more neocortex-based) feature more prominently. This is somewhat unclear as to why memory fragments appear more during REM, as opposed to there being merely fewer episodic references, however the switch in flow between hippocampus and neocortex could in part give rise to the interrupted and bizarre activation of memory fragments. Similarly, the increase in levels of cortisol across the night, peaking in the early hours of the morning, may also underlie the increasing levels of bizarre across the night (Payne, 2010).

As AM is multi-faceted, comprising both semantic and episodic elements (Renoult et al., 2012), it likely relies on consolidation from a full night of sleep and perhaps even and several iterations of sleep cycles over many nights. Furthermore Paller and Voss (2006) emphasize the cross-cortical storage across both sleep and wake, of declarative memory systems. This serves as a reminder of the complexity of both the memory and sleep systems involved in the encoding, consolidation, retrieval and re-consolidation of the majority of autobiographical information that we encounter in our daily, and nightly, lives.

## Is Dreaming a Reflection of the Processes of Sleep-Dependent Autobiographical Memory Consolidation?

The field of dream science has begun to address the experiential aspects of sleep-dependent memory consolidation (e.g., Blagrove et al., 2011c; Desseilles et al., 2011; Horton and Malinowski, 2011; Payne and Nadel, 2004; Horton, unpublished). This may in part be due to the relatively challenging nature of manipulating AMs in the laboratory, compared with, for instance, episodic memories for word lists or images. It may also be due to the complexity of the AM system, comprising both episodic and semantic elements over time. Nevertheless the AM system does account in theory for the constructive and changeable nature of recollection of personal experiences over time, and largely falls in line with systems of consolidation and re-consolidation, as described above.

There exists only a small body of empirical work on which to base the theory that dreaming reflects AM consolidation during sleep. We have already briefly summarized the trends depicting the incorporation of AMs into dreams (see Constructive Memory). However, many of these studies have investigated the memory sources of systematically-sampled dreams from a sleep-lab, at specific points in time. A fuller picture of the time-course of sleep-dependent consolidation can be sought by exploring the time between experiencing something in waking life and subsequently dreaming of it.

There are two broad trends: the day residue effect, which reflects the tendency to dream about experiences that occurred during the previous day and occurs in 65–70% of dreams (Nielsen and Powell, 1992), and the dream-lag effect (Nielsen, 2004; Nielsen et al., 2004; Blagrove et al., 2011a,b; van Rijn et al., 2015), which accounts for the tendency to dream about experiences that took place around a week (5–7 days) prior to the dream. The factors by which the dream-lag effect is produced are beginning to be identified, with REM-sleep providing the most reliable environment (Blagrove et al., 2011a) and memory salience increasing the likelihood of circaseptan (i.e., approximately week-long) incorporation (van Rijn et al., 2015). Explanations of dreaming, and in particular dreaming of specifically-aged experiences, are thus far largely only hypothetical (see discussions of Wamsley et al., 2010a; Murkar et al., 2014; Wamsley, 2014; van Rijn et al., 2015) and none yet have managed to account for the specific time-course of sleep-dependent memory consolidation processes. In part this reflects the variability in the time-course of setting down a memory into a specific, stable and long-term memory structure, as well as the complex nature of measuring this.

Nevertheless Marr (1970) proposed that consolidation rhythms guide the hippocampal-dependent store of the day’s events, which maps on to day residues appearing in dreams. Similarly, the circaseptan rhythms of consolidation (see Frankland and Bontempi, 2005) might reflect the comparably circaseptan dream-lag effects. However, the resurgence in dream-lag explorations are bringing about a fuller picture of the factors affecting incorporation of AMs into dreams, even if there is as yet no empirical evidence either that memory consolidation takes a week for salient experiences, or that these processes reflect the dream-lag effect directly or indirectly.

An alternative method of exploring the relationship between dreaming and sleep-dependent memory consolidation is that dreaming trends could map on to specific effects of memory enhancement following sleep. For instance, the increased likelihood of dreaming of experiences relevant to waking life during slow-wave sleep as opposed to REM (Baylor and Cavallero, 2001) aligns with the tendency for episodic memory gains to be associated with slow wave sleep rather than REM (Rauchs et al., 2004; Smith, 2004). Similarly, Dumel et al. (2015) have recently found that infrequent dream recall is associated with low performance but high overnight improvement on a procedural (mirror-tracing) task. This mode of investigation attempts to link dreaming behaviors with consolidation trends. One challenge of this method is to demonstrate the distinction between being able to recall or report a dream, and actually dreaming. That is, any measure of dreaming needs to be validated somehow. Additionally, could one dream because they learn better, or learn better because they dream?

Two additional studies (de Koninck et al., 1990; Miller and Horton, unpublished) have attempted to link directly the content of consolidated information in sleep with dream content. de Koninck et al. (1990) attempted to correlate the proficiency of learning a new language with dreaming of that new language, finding that individuals (in a sample of four) who improved their language learning incorporated the new language into their dreams earlier and more extensively than those who made little progress. This design has been extended by de Koninck’s team in a range of studies and makes use of language incorporations as a clearly operationalized and measurable mode of memory/learning incorporation into dream mentation.

The study by Horton involved exploring links between daily autobiographical experiences and dream content, finding that AMs that were incorporated into dreams were better recalled 3 weeks later than AMs that had not been incorporated into dreams. One criticism of this methodology is the difficulty in deciphering whether dreaming reflects memory consolidation, or whether memory improvements can be documented as a result of recording dream experiences which comprise a consolidated AM, thus activating and rehearsing those AMs in the process. However, indices of rehearsal were recorded and controlled for, and effects of memory improvement in line with dream trends remained. Nevertheless, methodological challenges still exist in this field (see A Model of AM Consolidation).

Overall empirical work attempting to link dreaming with memory consolidation is gaining momentum. Concurrently theoretical accounts of the relationships between memory fragmentation, hyperassociativity and memory consolidation are gaining prominence. Llewellyn (2013; Llewellyn and Hobson, 2015) has proposed one such theory, in which a function of dreaming of fragmented memory sources is to activate association networks, which lead to “elaborative encoding” and subsequent specific enhancement of episodic memory. Llewellyn’s theory recognizes that dreams contain fragmented AMs and that those fragments are synthesized into a new image comprising hyperassociated fragments, with links being activated during sleep between elements that would not typically be linked during wake. Llewellyn proposes several novel ideas, such as the mechanisms by which hyperassociations are “junctioned” within the hippocampus, and that the increased activation of association networks leads to elaborative encoding. Similarly, Wamsley (2014) has been pioneering in her championing the case for considering dream content in sleep-dependent memory research.

We step away from the emphasis on episodic memory, noting that the formation of unitary dream images comprising memory fragments from perhaps several different experiences from waking, are not vastly different from the construction of AMs in waking life in the self-memory system (Conway, 2009). Thus we instead see the AM system as a guide for the construction of memory fragments into unitary images across both sleep and wake (indeed all stages of sleep, albeit operating under slightly different conditions over the sleep–wake cycle).

## A Model of AM Consolidation

We have thus far reviewed the evidence for the involvement of AM across sleep and wake, noting that models of AM emphasize the re-construction of fragmentary pieces of information and experiences at recall. The continuity of the AM system across sleep and wake, largely measured by dream studies, highlights that AM should be acknowledged in models of sleep-dependent memory consolidation. Here we emphasize not only that AM can elucidate memory consolidation processes, but also that AMs can benefit from sleep-dependent consolidation.

**FIGURE 1:**
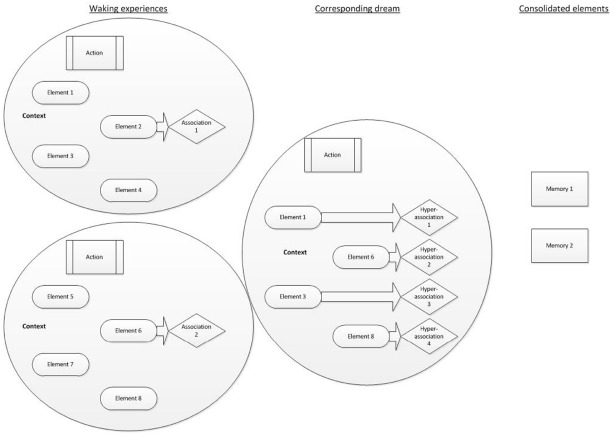
**AM consolidation model. Waking experiences are schematically represented as a circle, each containing numerous constituent fragments.** Individual *elements* (such as characters, colors, objects present, sounds, etc.) feature alongside an *action* (such as shouting at a colleague) and a *context* (such as the environmental setting and an emotional tone). Focused waking cognition involves the selective activation of semantic *associations* to some of the fragments. The **corresponding dream** involves the synthesis of different fragments (elements, actions and a context, plus their possible associations) taken from previous waking experiences. Each of those fragments could elicit a *hyperassociation*. The **consolidated elements** are those that have been repeatedly or strongly activated. They remain preferentially accessible following sleep.

We therefore propose a model of AM consolidation during sleep, which is depicted schematically in Figure 1.

As events are experienced, they contain several multi-modal elements, which can each be measured and broken down. Such elements may reflect units or features, such as characters involved, details of the setting, sounds or conversations, actions, colors, emotions, thoughts or other sensory experiences. A feature of waking cognition is focussed thought (e.g., Hartmann, 2010a,b). As such whilst semantic associations are activated during waking, executive functions largely ensure that resources are allocated to goal-directed tasks. Experiences, information and perceptions from waking are broken down into unitary elements and activated offline during subsequent sleep. Each element activates associates, which may be somewhat distantly-related to the original element via hyperassociativity. Dreams reflect the activation of these elements and their associates. Furthermore dreaming cognition, in contrast to focussed-waking thought, lacks goal-directed control. Coupled with hyperassociation, semantic associations may be activated and take over consciousness, leading to a loose, changeable and unfocussed stream of activated memories, thoughts and perceptions, in part as a result of the attenuation of executive functions during sleep (e.g., Maquet, 2000). The activation of disparate memory elements, hyperassociates, and their re-combination into novel permutations gives rise to the relatively bizarre and improbable events within single dream images. Repeated activation of elements during sleep, typically depicting recently-learned information or experiences, is widely considered to result in the consolidation of the activated elements (Born and Wilhelm, 2012).

The novelty of this model is that (i) dreaming directly reflects the processes of sleep-dependent memory consolidation, (ii) memories or information can be consolidated if merely related to an experience (via hyperassociativity), rather than being directly experienced, (iii) AM provides an account of the engagement of the whole declarative memory system, as opposed to necessitating episodic and semantic memories to be consolidated via different processes, and (iv) the fragmentation of AMs during sleep serves the function of decontextualizing them, rendering salient elements more easily integrated into existing networks, thus consolidating them efficiently.

Waking experiences are presented along with few associated thoughts and information, indicating a small degree of associated activation within focused, waking cognition. The consequential dream experience incorporates elements of the waking experiences, along with some new information and thus comprises novel permutations of the original AMs. Each dream element leads to activation of hyperassociated information. The subsequently consolidated features are those that have been repeatedly activated. These features may have been experienced in waking, in the dream or as associations of any element of these. There is therefore overlap between the original experiences, dreams, and consolidated features, in line with the constructive AM system. Figure 1 presents a schematic of these processes.

We can provide illustrations of how this may work using related waking and dreaming thoughts, as depicted in Table 1. The child’s dream exemplifies the typical emotionality of recalled dreams and how procedural and episodic elements can be combined within dream content, leading to independently consolidated features.

**TABLE 1:** **Application of the autobiographical memory consolidation model: child’s dream**.

**Waking experience**	**Associated dream**	**Consolidated features**
(i) Watching *Monsters Inc*. film in which monsters scare children in their bedrooms. Watching from my sofa in my living room, with baby sister and mother. This activates associated thoughts of scary creatures, such as a dragon from a favorite story book. (ii) Sat on Grandma’s knee in her chair, playing a new game on Grandma’s tablet. The object of the game is to fit puzzle shapes into a template. It makes a noise when the correct pieces are placed. This activates associated thoughts of photographs on the tablet, Grandma’s cats (sat nearby) and feelings of mastery when I accomplish the puzzle.	A monster (like the main character in the film) scares me in my bed. I am very frightened. I want to leave but I have to complete a puzzle to escape. I have to try several times but I can’t fit the last piece. I wake up just as the monster reaches me. This activates several hyperassociated elements such as being afraid of other specific film characters (who are usually friendly), being lost inside the tablet and seeing various shapes (within the puzzle).	Ability to fit the puzzle pieces together. Monsters can be scary.

The dream report has been taken from a 2.5-year-old child. The waking experiences have been provided by the child’s mother and the hyperassociated elements are hypothesized.

Table 2 presents an adult dream, depicting more mundane experiences and thoughts from daily life, reflective of more systematically-sampled dream content. The dream relates to the two waking life experiences and merges features of them, whilst individual elements of the waking life experiences have been broken down. The consolidated features arise from the repeatedly activated elements of the waking life experiences, the dream and the hyperassociations of the dream elements.

**TABLE 2:** **Application of the autobiographical memory consolidation model: adult’s dream**.

**Waking experience**	**Associated dream**	**Consolidated features**
(i) Sat at the computer at my desk in my office, preparing for an upcoming presentation on memory to a non-specialist audience. Trying to think of examples of procedural memory that people can relate to. People talking in the corridor nearby. This activates associated thoughts of colleagues in the field, the organizer of the presentation event, and other work projects that require urgent attention. (ii) Listening to the radio whilst washing up in the kitchen. A song by a well-known artist is being played, about a woman who escapes the daily hassles of her family and work by getting on a train. This activates associated thoughts of the singer’s husband (also well-known), own family commitments (needing to stock up on food to prepare better meals for the children) and work projects looming, such as the presentation described above.	Delivering a presentation (from (i)) on memory in a generic sports hall (an exam location) to people sat in rows behind small examination desks. The invigilator is the singer’s husband (from (ii)). I try to pronounce a brain region but falter several times. The scene shifts to a busy, unfamiliar road. I’m walking along it, discussing my shopping list with the singer’s husband again. We walk past his wife and wave. Several hyperassociated elements are activated, such as examination fears of nervousness, lack of preparation, brain regions I’ve been reading recently and images of the (unfamiliar) authors of related papers, hustle and bustle from the busy road, a memory of a newsagents from childhood, thoughts about my baby daughter’s ability to wave when she recognizes someone…	Representation of the film character consolidates recognition of their face and voice. A need to shop for food for the children. Examples of procedural memory needed for the presentation. A need to prepare the presentation so to avoid being nervous, and faltering. (The emotional context has been removed.)

The waking experiences and the dream report have been taken from the first author’s own experiences.

## Predictions of the Model

The AM consolidation model emphasizes the continuity between AM and dreaming cognition, as such there should be more similarities than differences between the activation of memories and thoughts dreaming and waking. In addition a major testable claim of our model is that the more something appears in a dream, the better consolidated and integrated into existing networks that should be.

As dreaming depends crucially on AM, those with AM impairment should also see a reduction in dreaming and impairments in consolidation. We believe that these propositions fit with standard models of both retrograde (with respect to the former) and anterograde (with respect to the latter) amnesia. Furthermore retrograde amnesics should be able to dream, but would likely only dream of recent experiences, if their accessibility of more remote memories is impaired across sleep as well as wake. Anterograde amnesics should likely only dream of older experiences, though there may well be methodological challenges with sampling dream reports from such patients.

We acknowledge the methodological challenges associated with testing some of these claims but recognize that paradigms are developing that aim to demonstrate links between dream content, whether direct or metaphorical representations are incorporated, and subsequent consolidation (Horton, unpublished data; Wamsley et al., 2010b).

## Methodological Note Concerning Sleep vs Dreaming

We wish to emphasize that we are not proposing that dreaming, *per se*, is a necessary and sufficient part of the memory consolidation process. Rather dreaming may reflect the processes of offline memory activation that leads to consolidation. Any mental content during sleep can give rise to the experience of having dreamt, providing waking occurs soon after and interference does not occur (Parke and Horton, 2009).Whilst these hypotheses are compelling, the data presented are largely preliminary and the field is still expanding. In particular the links between the sleep mentation and the nature of what exactly is being consolidated needs to be further refined. Nevertheless these data imply that there is something predictable about the nature of the sleep–wake cycle in relation to memory consolidation function. Animal studies are clearer here, although a review of these go beyond the scope of the present paper (however for a useful summary see Ribeiro and Nicolelis, 2004). Data from experimental studies also suggest that there is more continuity than discontinuity between memory functioning and consciousness across sleep and wake (e.g., Schredl and Hofmann, 2003). Similarly, some preliminary data has hinted that the content of daydreams can be directly linked to the subsequent improvement in a procedural dart-throwing task (Miller and Horton, unpublished data). These data provide evidence of the continuity of mental content as a reflection of procedural learning. The challenge is to integrate a theoretical explanation for this with the wealth of evidence underpinning current conceptions of sleep-dependent memory consolidation processes, which typically see sleep as providing an environment that differs substantially from waking functionality. It is essential to operationalize dreaming more specifically in order to enable predictions to be made about the continuity of cognitive processing across sleep and wake (Schredl and Hofmann, 2003).

We should also reiterate that we assume that the incorporation of AMs into dreams reflects the activation of AMs within the sleeping brain in a generally direct manner. That is, if something appears in a dream we deem it to be representative of some associated waking life experience. Whilst these manifestations may appear metaphorically or in a loosely associated manner, we should acknowledge that there exists some alternative theories of dreaming, which postulate that the representation of waking experiences into dreams are a compensation for the lack of conscious consideration during wake (e.g., Jung, 1934, 1948a,b). Freud also argued for dream content being far less reflective of waking life in his theory of repression.

## Individual Differences, Implications for Creativity, and Clinical Considerations

The hyperassociative cognition typical of REM sleep can promote creativity and insight (e.g., Ritter et al., 2012). Yet disturbed sleep can lead to greater dissociation, also resulting in greater creativity (van Heugten-van der Kloet et al., 2015). How can these seemingly opposing views be aligned?

Disturbed sleep is recognized as a correlate of most diagnosable mental illnesses, both psychotic and neurotic/emotional in nature. As some individuals show a tendency toward creativity in waking it would follow, in line with the continuity hypothesis and its emphasis on holistic functioning over the full cycle of sleep and wake, that some individuals may be particularly affected by sleep’s hyperassociative cognition. Some researchers have investigated these ideas with regards to creativity and psychosis (e.g., Llewellyn, 2011). In non-clinical contexts, “healthy” sleep can promote creativity in relation to problem solving, as novel insight results from hyperassociative cognition during sleep (Stickgold et al., 1999). When sleep is more disturbed dissociation can result, which in turn can lead to creative thinking. This creates a U-shaped relationship between sleep quantity and creativity. The question therefore remains as to how this may affect memory consolidation.

One could argue that the greater the fragmentation of autobiographical experiences during sleep, resulting in part from hyperassociative cognition, the greater the experience of dream bizarreness and subsequent creativity in waking. Such processing would also lead to greater de-contextualization of a remembered memory source, subsequently increasing its retrievability across different situations. According to our view this reflects an efficient mode of AM consolidation, resulting from obtaining non-disturbed sleep.

We propose that disturbed sleep would lead to impaired AM functioning. As AM comprises both memories about one’s own life as well as a cognitive self, we hypothesize that disturbed sleep would impact on both the retrievability of AMs as well as the stability of the self.

## Conclusion

We have presented a model of AM functioning across sleep and wake, such that memory sources of autobiographical experiences are fragmentarily activated during sleep and re-bound with other memory sources, as manifested via bizarre combinations of experiences in dreams. The rebinding of memory sources occurs when the brain enters a hyperassociative, reflexive state during both REM and non-REM sleep. Such processes are proposed to be reflective of sleep-dependent memory consolidation. There are therefore benefits of such hyperassociative and creative cognitive processing, as under the right conditions, they can lead to de-contextualization of experiences and subsequent increased accessibility of a memory.

This is the first time that AM, engaging both the semantic and episodic (declarative) memory systems, has been proposed to account for the activation of hippocampal–cortical regions during sleep. We argue that studying the composition of dreams provides a useful methodological tool for further exploring the cognitive processes of sleep-dependent memory consolidation.

### Conflict of Interest Statement

The authors declare that the research was conducted in the absence of any commercial or financial relationships that could be construed as a potential conflict of interest.
